# Pine monoterpenes drive growth, competition, and volatile metabolism of symbiotic fungi associated with the mountain pine beetle

**DOI:** 10.1093/femsle/fnag096

**Published:** 2026-08-26

**Authors:** Nadya Citra, Yanzhuo Liu, Guncha Ishangulyyeva, Nadir Erbilgin

**Affiliations:** Department of Renewable Resources, University of Alberta, Edmonton, Alberta T6G 2E3, Canada; Department of Renewable Resources, University of Alberta, Edmonton, Alberta T6G 2E3, Canada; Department of Renewable Resources, University of Alberta, Edmonton, Alberta T6G 2E3, Canada; Department of Renewable Resources, University of Alberta, Edmonton, Alberta T6G 2E3, Canada

**Keywords:** terpene-mediated resistance, volatile signalling, metabolic tolerance, fungal pathogenicity, secondary metabolites, host–symbiont interactions

## Abstract

Monoterpenes are key conifer defences that shape interactions between the mountain pine beetle and its symbiotic phytopathogenic fungi. Conifer monoterpenes vary both within and between tree species, yet how this variation affects symbiotic fungi remains poorly understood. We tested how monoterpene blends from three geographically distinct subspecies of *Pinus contorta*, including subsp. *contorta, latifolia*, and *murrayana* can affect the growth and volatile emissions of two fungal symbionts, *Grosmannia clavigera* and *Ophiostoma montium*, of the mountain pine beetle. We conducted a factorial experiment using monoterpene blends representing the three subspecies in combination with three fungal cultures (two alone and one mixed) to quantify fungal biomass and volatile organic compounds. Monoterpene blends reduced fungal biomass, with the subsp. *murrayana* blend is the most inhibitory *G. clavigera* tolerated monoterpenes better than *O. montium*, suggesting enhanced virulence. Fungal volatile emissions varied by blend and culture; 2-methyl-1-butanol and isobutanol were positively correlated with growth, whereas verbenone and *cis*-grandisol were negatively correlated. These findings show that the monoterpene diversity of host trees differentially modulates fungal growth and volatile emission, revealing chemical mechanisms underlying host colonization by the mountain pine beetle across regions.

## Teaser

Natural monoterpene blends in lodgepole pines from different regions can suppress the symbiotic fungi of mountain pine beetles and alter their odours, revealing chemical clues to why some forests are more vulnerable.

## Introduction

Monoterpenes are small, volatile hydrocarbons that represent the largest class of plant terpenes and function as primary chemical defences against pests and pathogens (Phillips and Croteau 1999, Trapp and Croteau 2001, Keeling and Bohlmann 2006). They typically occur as complex blends of structurally diverse compounds, with compositions varying among plant species and across climatic regions due to genetic and environmental factors (Smith 1967, Sampedro et al. 2011, Moreira et al. 2014, Celedon and Bohlmann 2019). In conifers, monoterpenes are major components of oleoresin that mediate interactions with one of their most significant forest insects, bark beetles (Coleoptera: Curculionidae, Scolytinae), and their associated fungi (Keeling and Bohlmann 2006, Erbilgin et al. 2007, Raffa et al. 2008, 2013, Erbilgin 2019, Liu et al. 2021, Raffa and Berryman 1983a, b, Wang et al. 2020a, Seidl et al. 2011, Ullah et al. 2021). These compounds are toxic to beetles and fungi, deterring them from the tree. In response, beetles have coevolved with symbiotic fungi that help them overcome host defences and enhance colonization success (Raffa et al. 2008, Six 2012, Zhang et al. 2026). Importantly, monoterpenes not only affect fungal growth but also their communication with beetles (Wang et al. 2020a, Cale et al. 2019, Wang et al. 2019), thereby enabling host defences to indirectly influence beetle behaviour through fungus-mediated chemical signalling (Kandasamy et al. 2016). Understanding how monoterpenes regulate fungal growth and communication is, therefore, critical to unravelling the chemical ecology of beetle infestations.

Lodgepole pine (*Pinus contorta*) is a dominant conifer of the western North American boreal forest, exhibiting a wide climatic range. Its distribution extends from Alaska (USA) and the Yukon (Canada) in the north to Baja California (Mexico) in the south, occupying diverse environments including the Rocky Mountains, Pacific coastal regions, and inland areas of British Columbia and Alberta (Canada) (Critchfield 1957, Lotan and Critchfield 1990, Millar and Rundel 2016). This species produces over 25 distinct monoterpenes, with compositions and relative concentrations varying significantly across its range. Such chemical variability reflects the species’ adaptability to heterogeneous environments and divergent selective pressures (Forrest 1980, Raffa et al. 2017, Erbilgin 2019, Liu et al. 2025).

A major selective pressure on lodgepole pine is the mountain pine beetle (*Dendroctonus ponderosae* Hopkins, MPB), whose distribution largely overlaps that of its host tree species (Safranyik and Carroll 2006). Mountain pine beetle outbreaks have caused extensive ecological and economic damage across western North America, resulting in widespread tree mortality and disruption of forest ecosystem services (Raffa et al. 2008, Safranyik et al. 2010). Importantly, tree susceptibility to MPB varies spatially and is influenced by local climate, host chemistry, and the beetle’s interactions with its fungal symbionts (Zaman et al. 2023a, b, Cale et al. 2017, Erbilgin et al. 2017, Ullah et al. 2021, Agbulu et al. 2022).

The MPB is closely associated with two major ophiostomatoid fungi (Ascomycota: Ophiostomataceae), *Grosmannia clavigera* and *Ophiostoma montium*, which are transported *via* specialized beetle structures called mycangia (Bleiker et al. 2009). These fungi assist beetles by detoxifying host defences and necrotizing host tissue while benefiting from access to host nutrients (Wang et al. 2013, 2014, Zaman et al. 2023a, b). *Grosmannia clavigera* is notable for its virulence and ability to detoxify host defences, whereas the slower-growing *O. montium* functions primarily as a nutrient source (Rice et al. 2007a, Lah et al. 2013, Wang et al. 2013, 2014, Chiu et al. 2019, Guevara-Rozo et al. 2020).

Several monoterpenes exhibit strong antifungal activity, such as α-pinene, 3-carene, limonene, and myrcene (Cale et al. 2017, Reid et al. 2017, Ullah et al. 2021, Zhang et al. 2026). Other pine volatiles, such as 4-allylanisole, are even more toxic, demonstrating inhibitory effects at very low concentrations (Ullah et al. 2021, Zhang et al. 2026). Interestingly, individual monoterpenes often exert opposite effects on fungi and the MPB, with compounds highly toxic to fungi being less harmful to beetles (Chiu et al. 2017, Wang et al. 2019, Ullah et al. 2021). Lodgepole pine appears to have evolved synergistic monoterpene blends that provide a multifaceted defence system effective against both threats (Ullah et al. 2021, Zaman et al. 2025). However, it remains unclear how these blends differentially affect beetles and their fungal symbionts. Despite the critical role of fungal partners in successful host colonization, most research has focused primarily on beetle responses to monoterpene blends (Erbilgin et al. 2017, Erbilgin 2019), underscoring the need to examine how these compounds influence fungal growth and volatile emissions.

Fungal associates also produce their own fungal volatile organic compounds (FVOCs), which function as semiochemicals that modulate beetle behaviour (Cale et al. 2016, 2019). Beetles use these cues to locate suitable hosts and regulate aggregation or dispersal (Zaman et al. 2023b, Kandasamy et al. 2023). Beyond their effects on beetles, fungal volatiles mediate interactions among fungal species, influencing growth and/or conidia production (Wang et al. 2020b, Cale et al. 2016). The composition and relative concentrations of these FVOCs can shift in response to host monoterpenes (Hung et al. 2015, Cale et al. 2019), thereby indirectly modifying beetle–fungus interactions through changes in fungal signalling (Kandasamy et al. 2016). Collectively, these reciprocal chemical exchanges between lodgepole pine and fungi likely regulate MPB colonization dynamics, as observed in other conifer–beetle–fungus systems (Agbulu et al. 2022, Cale et al. 2025).

Understanding how host monoterpene blends regulate fungal growth and volatile emissions is therefore essential to elucidate the chemical foundations of beetle infestations. Furthermore, accounting for the substantial variation in lodgepole pine chemistry across climatic regions may help explain spatial patterns in susceptibility to MPB attack (Forrest 1980, Erbilgin 2019, Ullah et al. 2021, Liu et al. 2025, Han et al. 2026).

This study investigated how monoterpene blends from lodgepole pine across varying climatic regions influence the growth, volatile emissions, and interactions of *G. clavigera* and *O. montium*. We focused on monoterpene blends representative of three lodgepole pine subspecies: subsp. *contorta* (British Columbia), subsp. *latifolia* (Alberta), and subsp. *murrayana* (Oregon). Specifically, we addressed the following research questions: (1) Do different monoterpene blends differentially affect fungal growth across single-species and mixed-species cultures? (2) Do monoterpene blends differentially influence FVOC emissions across single-species and mixed-species cultures? (3) Do variations in fungal growth correspond with the changes in FVOC emissions under varying monoterpene blends?

## Materials and methods

### Experimental design

We obtained isolates of *G. clavigera* (EL004) and *O. montium* (EL031) from MPB galleries in lodgepole pine collected by N. Erbilgin in Banff (May 2016) and Westcastle (January 2015), Alberta, Canada, respectively. Master cultures of both isolates were prepared on potato dextrose agar and incubated in complete darkness at 22°C for 10 days, adjusted based on the general liquid-culture exposure method described by Cale et al. (2019). We excised agar plugs from the actively growing margins of 10-day-old master cultures and inoculated them into 30 ml of sterile potato dextrose broth (PDB) in 50 ml Erlenmeyer glass flasks for monoterpene exposure experiments. To minimize monoterpene volatilization, the liquid media were allowed to cool to room temperature (20°C–22°C) before monoterpene amendment. After monoterpenes were added, the flask openings were wrapped twice with Parafilm M laboratory film (Amcor, Neenah, WI, USA), and the cultures were incubated without shaking in complete darkness at 22°C for 8 days.

We used a full-factorial design comprising five treatments (three different monoterpene amendments, one ethanol-only control, and one negative control) crossed with three fungal cultures (two single-species inoculations and one mixed-species inoculation), resulting in 15 treatment combinations. Each treatment was replicated 10 times.

We developed three monoterpene amendment treatments based on the average phloem monoterpene concentrations of three subspecies of *P. contorta*, collected from three locations: *P. contorta* subsp. *latifolia* (Hinton, Alberta, Canada; 53.3378° N, 115.4861° W), *P. contorta* subsp. *contorta* (Smithers, British Columbia, Canada; 55.44° N, 126.68° W), and *P. contorta* subsp. *murrayana* (Bend, Oregon, USA; 43.687988° N, 121.276258° W). The amendments were composed predominantly of monoterpenes but also included 4-allylanisole, a phenylpropene naturally present in lodgepole pine phloem. Monoterpene concentrations were calculated from ng mg^−1^ fresh weight of phloem tissue (Table S1) and converted to μl ml^−1^ liquid medium to simulate natural phloem monoterpene concentrations (Table S2).

We prepared monoterpene stock solutions using the following standards: (–)-α-pinene (chemical purity: 98%), (+)-α-pinene (98%), (–)-β-pinene (>94%), (+)-β-pinene (≥98.5%), (–)-limonene (96%), (+)-limonene (97%), (–)-camphene (75%), (+)-camphene (≥80%), 3-carene (≥95%), linalool (97%), α-terpinene (95%), γ-terpinene (97%), terpinolene (≥85%), 4-allylanisole (≥98.5%), (+)-pulegone (97%), *p*-cymene (99%), bornyl acetate (≥99%), (–)-borneol (≥99%), α-phellandrene (≥95%), ocimene (≥90%), myrcene (≥75%), sabinene hydrate (≥97%), camphor (96%), α-terpineol (90%), and thujone (≥96%) (all from Sigma-Aldrich, St. Louis, MO, USA). Ethanol was used as the solvent to dissolve the monoterpenes. For each treatment, we dissolved 300 μl of monoterpene stock solution into the 30 ml PDB liquid media (corresponding to 1% v/v). Two control treatments were also included: first with 300 μl of 100% ethanol and second with no amendments.

We tested three fungal cultures: *G. clavigera* alone, *O. montium* alone, and a combination of both. Each treatment received two fungal plugs to maintain a consistent number of inoculated plugs across the three cultures. For single-species cultures, both plugs were from the same species. For the mixed-species cultures, fungal biomass was measured as the total combined dry biomass of both fungi, with species-specific abundance not quantified.

We adapted the method described by Cale et al. (2019) to quantify fungal biomass. Briefly, after the 8-day growth period, we collected 4 ml of hyphae-free liquid from each culture flask and transferred it into clean 8 ml glass tubes with caps for chemical analysis. The remaining culture broth was vacuum filtered through preweighed filter paper (Grade 1, Whatman, Maidstone, UK) to collect the fungal mycelium. Agar plugs used for inoculation were removed from the retained mycelium before the samples were lyophilized for 48 h (Labconco Corporation, Kansas City, MO, USA). After freeze-drying, the filter papers containing mycelium were reweighed, and fungal dry biomass was calculated as the difference between the weight of the filter paper with mycelium and the weight of the blank filter paper.

### Fungal VOC chemical analysis

We extracted FVOC compounds directly from the previously collected 4 ml of mycelia-free liquid broth by adding 1 ml of dichloromethane containing 0.001% tridecane as an internal standard. We vortexed the mixture for 30 s, centrifuged it at 7830 rpm for 15 min at 4°C, and transferred 1 ml of the organic phase to a 2 ml glass gas chromatography (GC) vial. We repeated the extraction once to obtain a total of 2 ml of extract, which we stored at –40°C until further analysis.

We analysed the FVOC extracts using a gas chromatograph (GC 7890A) coupled to a mass spectrometer (MS 5975C) (Agilent Technologies, Santa Clara, CA, USA). We operated the GC with a DB-5MS UI column (30 m length, 0.25 μm film thickness) and used helium as the carrier gas at a constant flow rate of 1 ml min^−1^. Each sample (1 μl) was injected in splitless mode. The oven temperature program started at 50°C (held for 1 min), increased to 200°C at a rate of 5°C min⁻¹, and then to 325°C at 30°C min⁻¹, where it was held for 2 min. We identified compounds by comparing their retention times and mass spectra with those of authentic analytical standards: acetoin (chemical purity: 95%), isoamyl acetate (95%), isobutanol (99%), 2-methyl-1-butanol (99%), 3-methyl-1-butanol (98%), phenethyl acetate (98%), and (–)-verbenone (94%), and *cis*-grandisol (98%). Each compound was quantified using a compound-specific calibration curve prepared from serial dilutions of its corresponding authentic standard. Tridecane was included at the same concentration in all calibration standards and sample extracts. Calibration curves were generated by regressing the analyte-to-internal-standard peak-area ratio against the known analyte concentration, and sample concentrations were calculated using the corresponding regression equation. Peaks detected in the unamended control samples were also excluded from the analysis.

### Statistical analysis

All following statistical analyses and graphic visualizations are performed in R (version 4.5.1) under macOS. To evaluate differences in fungal dry biomass across the 15 treatment combinations (five amendments *× * three fungal cultures), we applied a nonparametric Kruskal–Wallis test, as the treatment groups were independent and the data were not normally distributed. Following a significant result, we conducted *post hoc* pairwise comparisons using Dunn’s test with Benjamini–Hochberg correction to control for false discovery rate.

To examine variation in FVOC composition and relative concentrations across treatments, we applied Z-score normalization to the volatile compounds across the 15 treatment combinations. Column-wise normalization standardized the concentrations of each compound across treatments, facilitating comparison of treatment effects on individual compounds. Row-wise normalization standardized compound concentrations within each treatment, allowing comparison of overall volatile emission profiles among treatments. Hierarchical clustering was performed using Euclidean distance and complete linkage to group similar treatments or compounds, depending on the normalization direction.

To assess how monoterpene amendment and fungal culture jointly influenced the composition and relative concentrations of FVOCs in each treatment combination, we performed redundancy analysis (RDA), which is a constrained ordination method that combines multiple linear regression with principal component analysis. It extracts canonical axes that represent the linear combinations of explanatory variables (monoterpene amendments, fungus, and their interactions) that best explain the variation in multivariate response variables (the composition and relative concentrations of FVOCs across treatments). Statistical significance of the overall model, individual terms, and constrained axes was assessed using permutation tests with 999 iterations. To quantify the individual and interactive effects of monoterpene amendment and fungal culture on each volatile compound, we conducted two-way ANOVA separately for each FVOC. It partitions the total variance of each compound’s concentration into components attributable to the individual effects (monoterpene amendment or fungal culture) and their interaction (monoterpene amendment × fungal culture).

To examine pairwise associations between FVOCs and fungal biomass, we first applied Pearson correlation analysis to log-transformed quantitative variables. This approach estimates the linear correlation coefficient (r) between each variable pair, ranging from −1 to + 1. To further investigate nonlinear associations between FVOCs and fungal biomass, we fitted a generalized additive model using penalized regression splines. To limit model complexity and avoid overfitting, six representative FVOCs were selected as predictors based on the default upper limit on basis dimensions (k = 7) and to ensure monoterpene- and treatment-level diversity. The model included a smoothing term for each compound, with outputs reporting estimated degrees of freedom (edf), *F*-values, and corresponding *P*-values. Model performance was evaluated using adjusted *R*² and deviance explained. All FVOC concentrations were log-transformed prior to analysis to reduce skewness and stabilize variance.

## Results

### Monoterpene amendments differentially reduced the fungal biomass of *G. clavigera* and *O. montium*

**Figure 1:**
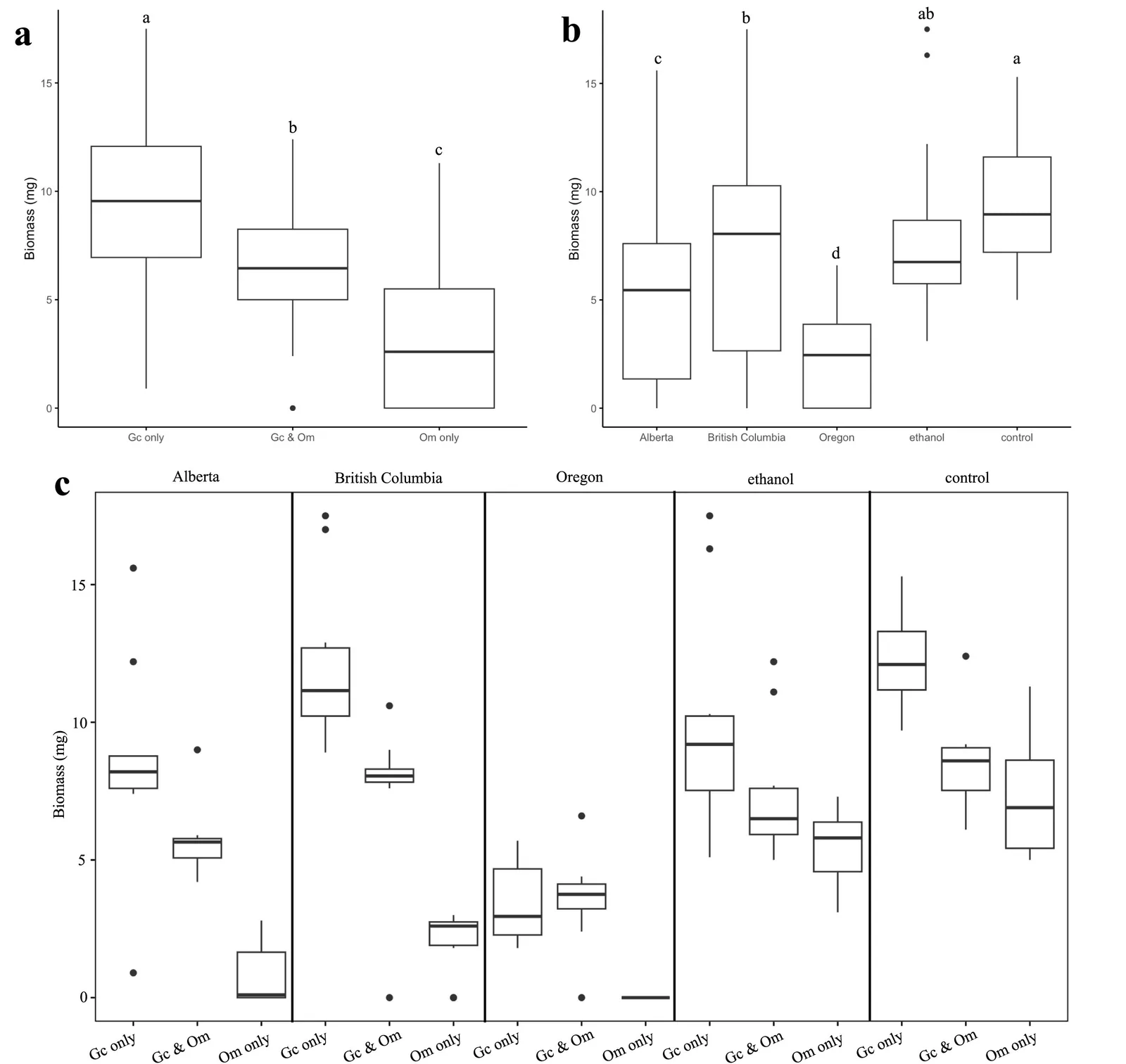
Effects of fungal treatment and monoterpene amendment on fungal biomass production. (a) Boxplot comparing fungal biomass (mg) among three fungal treatments: *G. clavigera* (Gc), *O. montium* (Om), and their combination (Gc/Om). (b) Comparison of biomass (mg) across five monoterpene amendments. (c) Interaction effects between fungal treatment and monoterpene amendments, with biomass (mg) distributions shown for each treatment combination grouped by monoterpene amendment. Different lowercase letters above boxes in (a) and (b) indicate statistically significant differences based on pairwise comparisons with adjusted *P* < .05. Results of multiple comparisons for panel (c) are provided in Table S3. Horizontal lines indicate medians; boxes represent interquartile ranges (IQR); whiskers extend to 1.5 × IQR; points denote outliers.

There were significant differences in overall fungal biomass across monoterpene amendments (Kruskal–Wallis test: χ² = 55.28, df = 4, *P* < .001; Fig. 1b), and the control treatment without any amendment had the highest overall fungal biomass. No significant difference was observed between the control and the ethanol-only amendment (Z = 1.89, *P* = .065). Biomass in British Columbia amendment was lower than in the control (Z = −2.15, *P* = .045), followed by Alberta (Z = −2.09, *P* = .046) and Oregon, which had the lowest biomass.

There were significant differences in *G. clavigera* and *O. montium* biomass within the same monoterpene amendments (e.g. Kruskal–Wallis χ² = 52.35, df = 2, *P* < .001; Fig. 1a), with *G. clavigera* consistently having higher biomass than *O. montium*. The same pattern was observed across all five amendments (Alberta: Z = 4.26, *P* < .001; British Columbia: Z = 5.35, *P* < .001; control: Z = 2.45, *P* = .034; ethanol: Z = 2.33, *P* = .042; Oregon: Z = 4.67, *P* < .001). Mixed species culture generally produced intermediate biomass, lower than *G. clavigera* but higher than *O. montium* in several cases.

There were also significant differences in fungal biomass across the 15 combination treatments (monoterpene blend *× * fungal culture) (Kruskal–Wallis test: χ² = 118.63, df = 14, *P* < .001; Fig. 1c; pairwise comparisons in Table S3). The highest biomass being *G. clavigera* under the control treatment, and the lowest biomass was *O. montium* of the Oregon amendment.

### Monoterpene amendments and fungal species affected FVOC emissions

**Figure 2:**
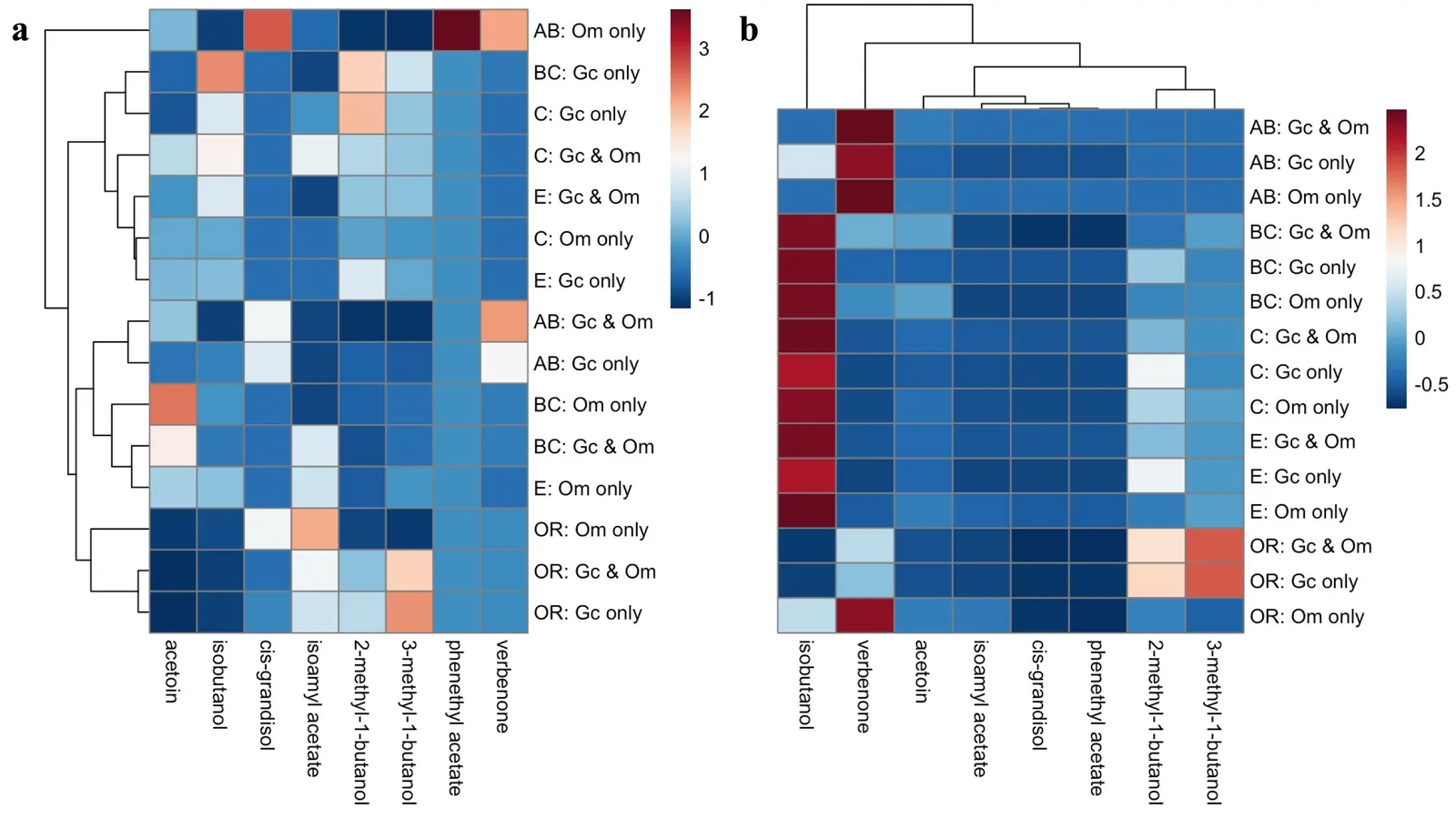
Heatmaps showing treatment-level variation in FVOCs based on Z-score normalization. (a) Compound-wise normalization was standardized across all treatments, allowing comparison of emission intensity among treatments for each compound. (b) Treatment-wise normalization was standardized across all compounds, highlighting the relative abundance of each compound within a treatment. Rows represent unique combinations of monoterpene amendments (AB: Alberta; OR: Oregon; BC: British Columbia; C: Control; and E: ethanol) and fungal treatment (Gc: *Grosmannia clavigera*, Om: *Ophiostoma montium*; and Gc and Om: *G. clavigera* and *O. montium* combination). Hierarchical clustering was applied to group similar treatments (a) or compounds (b). Colour scale indicates standardized abundance (red = high, blue = low).

Clustering compound-wise Z-score normalization revealed volatile compounds primarily grouped by monoterpene amendments (Fig. 2a). Two major branches emerged: one consisting solely of *O. montium* under the Alberta amendment, which formed a distinct cluster characterized by elevated concentrations of verbenone, phenethyl acetate, and *cis*-grandisol; and another, larger cluster encompassing the remaining treatments. Within this larger group, treatments involving *G. clavigera, O. montium*, or both did not separate clearly. Notably, treatments amended with the Oregon monoterpenes formed a well-defined subcluster marked by lower concentrations of most compounds (dark blue), except for relatively higher concentrations of 3-methyl-1-butanol, 2-methyl-1-butanol, and isoamyl acetate.

Clustering based on treatment-wise normalization highlighted compound-specific expression patterns across the 15 combination treatments (Fig. 2b). The first major branch indicated elevated concentrations of isobutanol in treatments with British Columbia amendment, no amendment (control), and ethanol-only amendment. The second main branch revealed elevated concentrations of verbenone in *O. montium* treatments under Alberta and Oregon amendments. The remaining compounds were emitted at low concentrations, forming two subclusters: one cluster was the emissions of 3-methyl-1-butanol and 2-methyl-1-butanol in *G. clavigera* and coculture treatments under Oregon amendment; and another was the emissions of acetoin, isoamyl acetate, *cis*-grandisol, and phenethyl acetate under all treatments.

**Figure 3:**
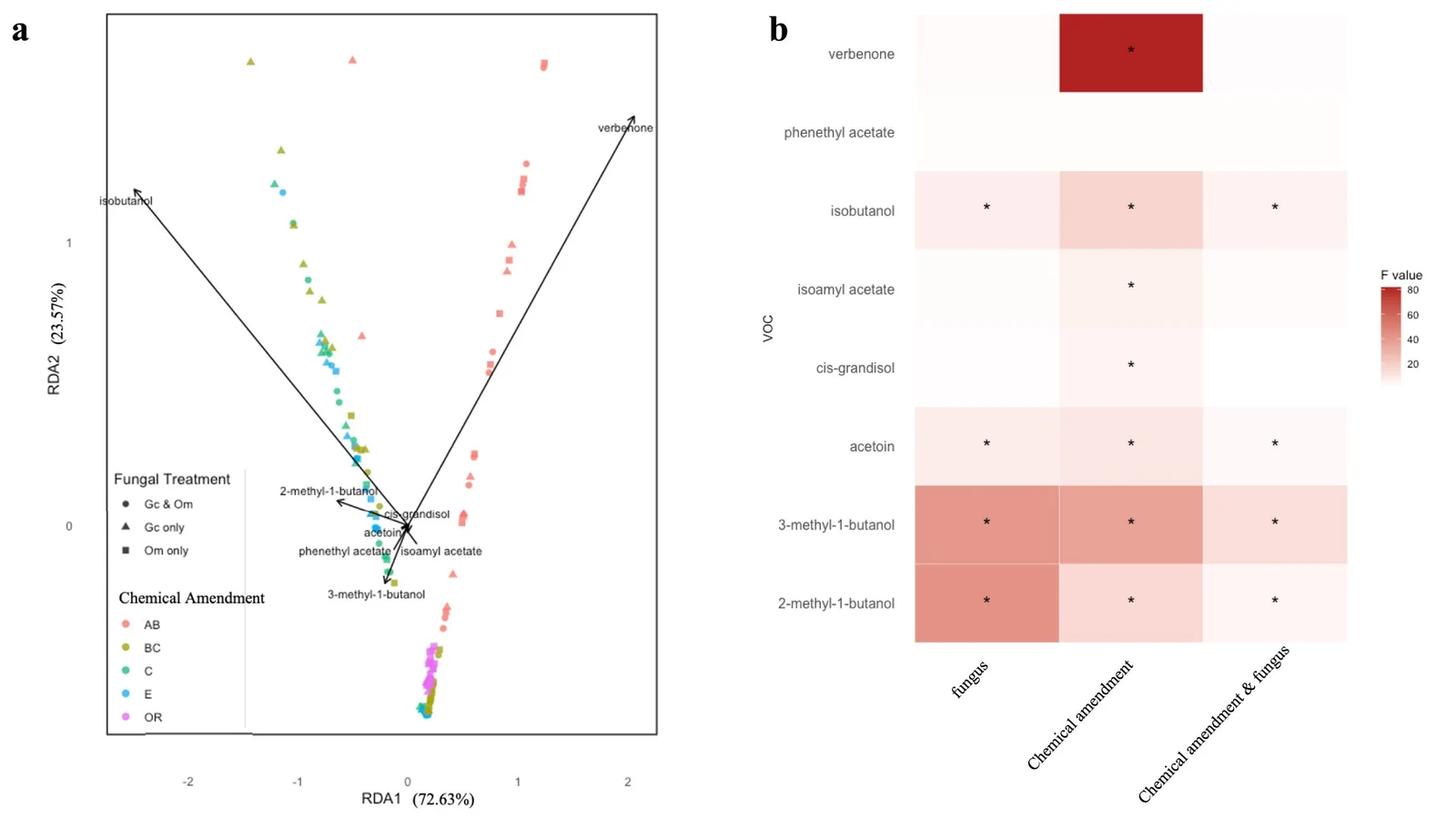
Effects of monoterpene amendment and fungal treatment on FVOC composition of *G. clavigera* (Gc), *O. montium* (Om), and the combination of Gc and Om. (a) RDA biplot showing the constrained ordination of samples based on volatile compound composition. RDA1 and RDA2 represent the first and second constrained axes, explaining 72.63% and 23.57% of the total constrained variation, respectively. Samples are colored by monoterpene amendments and shaped by fungal treatments. Arrows indicate the direction and strength of each FVOC’s contribution to the ordination space. (b) Factorial ANOVA heatmap showing *F*-values for each FVOC under three sources of variation: fungus, monoterpene amendment, and their interaction. Fill colour represents the *F* statistic, with darker red indicating stronger effects. Asterisks denote statistically significant effects (*P* < .05). Monoterpene amendments: AB = Alberta; OR = Oregon; BC = British Columbia; C = control; and E = ethanol.

RDA revealed that FVOC emissions were significantly influenced by both monoterpene amendment and fungal culture (*F* = 12.55, *P* < .001, Fig. 3a). RDA1 and RDA2 accounted for 96.2% of the variance (RDA1: 72.63% and RDA2: 23.57%). Permutation tests showed that monoterpene amendment (*F* = 31.14, *P* < .001), fungal culture (*F* = 7.83, *P* < .001), and their interaction (*F* = 4.43, *P* < .001) all contributed significantly to FVOC variation. Both RDA1 (*F* = 134.18, *P* < .001) and RDA2 (*F* = 43.55, *P* < .001) were significant axes.

Along RDA1, treatments were clearly separated by monoterpene amendments. Specifically, treatments amended with Alberta, Oregon, and some British Columbia monoterpenes clustered to the right, while ethanol, control, and other British Columbia treatments clustered on the left. Oregon treatments showed partial separation from other monoterpene amendments along RDA2. No consistent separation was observed among fungal treatments within the same monoterpene amendment, suggesting that monoterpene amendment had a stronger effect on FVOCs than fungal culture.

Consistent with clustering results, verbenone was strongly associated with Alberta amendment, whereas isobutanol was associated with ethanol and control. Moreover, 3-methyl-1-butanol, 2-methyl-1-butanol, and isoamyl acetate, clustered near the origin, showing weak associations with ethanol and control treatments.

Factorial ANOVA further confirmed that monoterpene amendment, fungal culture, and their interaction significantly affected FVOC emissions (Fig. 3b). Fungal culture affected five compounds, including isobutanol (*F* = 6.80, *P* = .002), acetoin (*F* = 7.73, *P* = .001), 3-methyl-1-butanol (*F* = 41.00, *P* < .001), 2-methyl-1-butanol (*F* = 42.79, *P* < .001), and isobutanol (*F* = 6.80, *P* = .002). Monoterpene amendment had a broader influence, significantly impacting six FVOCs, most notably verbenone (*F* = 82.05, *P* < .001), but also 3-methyl-1-butanol (*F* = 36.63, *P* < .001), 2-methyl-1-butanol (*F* = 15.48, *P* < .001), isobutanol (*F* = 17.01, *P* < .001), acetoin (*F* = 10.15, *P* < .001), isoamyl acetate (*F* = 5.93, *P* < .001), and *cis*-grandisol (*F* = 4.56, *P* = .002). Interactive effects of monoterpenes amendments and fungal culture were significant for 3-methyl-1-butanol (*F* = 13.14, *P* < .001), 2-methyl-1-butanol (*F* = 4.19, *P* < .001), isobutanol (*F* = 5.19, *P* < .001), and acetoin (*F* = 2.81, *P* = .006). In contrast, phenethyl acetate showed no significant response to any factor.

### FVOCs affected fungal biomass

**Figure 4:**
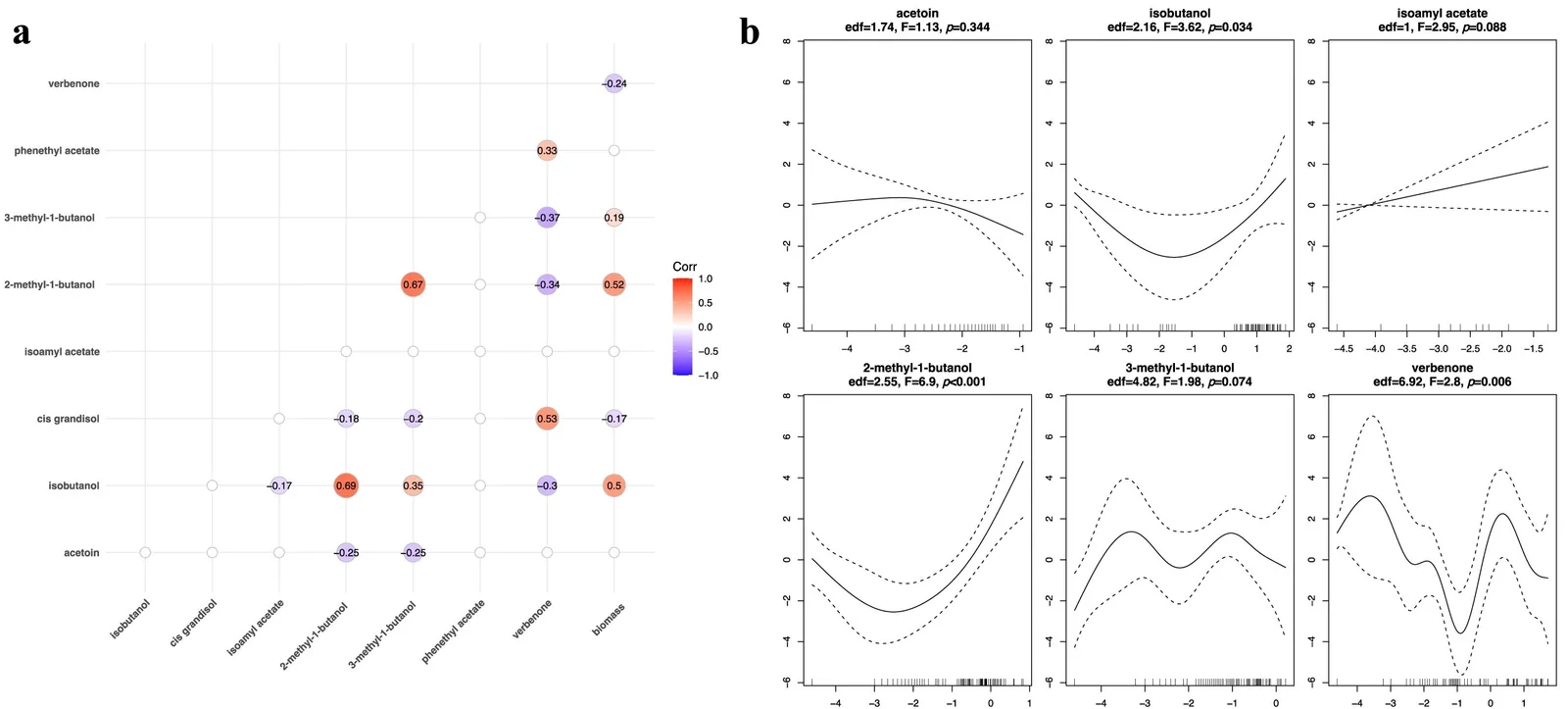
Correlation and nonlinear associations between fungal biomass and individual FVOC. (a) Pearson correlation matrix showing pairwise linear correlations between fungal biomass and individual FVOCs. Colour scale indicates the strength and direction of correlation (red: positive, blue: negative), and only statistically significant correlations (*P* < .05) are shown. Numbers within circles represent the Pearson correlation coefficient (r). (b) Generalized additive model plots illustrating nonlinear relationships between six representative FVOCs and fungal biomass. FVOC concentrations were log-transformed prior to modelling. The *x*-axis represents the log-transformed concentration of each FVOC, and the *y*-axis represents the centred and scaled effect of each VOC on fungal biomass (partial residual). Solid lines represent the fitted smooth terms, and dashed lines indicate 95% confidence intervals. Each panel reports the estimated degrees of freedom (edf), *F*-value, and *P*-value for the corresponding smoothing term.

Pearson correlation analysis revealed significant associations between FVOCs and fungal biomass (Fig. 4a). Both 2-methyl-1-butanol (*r* = 0.52, *P* < .001) and isobutanol (*r* = 0.50, *P* < .001) were also positively correlated with fungal biomass. Verbenone was negatively correlated with 3-methyl-1-butanol (*r* = –0.37, *P* < .001), 2-methyl-1-butanol (*r* = –0.34, *P* < .001), and isobutanol (*r* = –0.30, *P* < .001), and showed a significant negative correlation with biomass (*r* = –0.24, *P* = .003). Similarly, *cis*-grandisol was positively correlated with verbenone (*r* = 0.53, *P* < .001) and negatively associated with biomass (*r* = –0.17, *P* = .038).

To further investigate nonlinear relationships between individual FVOCs and fungal biomass, we applied generalized additive models using six key compounds as predictors (Fig. 4b). The model explained 60.3% of the deviance (*R*²_adj_ = 0.545), indicating a statistical fit. Among the smooth terms, 2-methyl-1-butanol exhibited the strongest and most significant positive association with biomass (edf = 2.55, *F* = 6.90, *P* < .001), with a sharp increase in biomass at higher concentrations. Verbenone also significantly influenced biomass (edf = 6.92, *F* = 2.80, *P* = .006), showing a nonmonotonic relationship, where biomass was lowest at intermediate verbenone concentrations. Isobutanol displayed a weak but significant U-shaped relationship with biomass (edf = 2.16, *F* = 3.62, *P* = .034), with reduced biomass at moderate concentrations. In contrast, 3-methyl-1-butanol showed a nonsignificant trend (edf = 4.82, *F* = 1.98, *P* = .074), with no clear directional pattern. Isoamyl acetate (edf = 1.00, *F* = 2.95, *P* = .088) and acetoin (edf = 1.74, *F* = 1.13, *P* = .344) did not affect biomass.

## Discussion

Lodgepole pine monoterpene blends exhibited varying degrees of toxicity towards *G. clavigera* and *O. montium*. Consistent with previous studies, all monoterpene blends inhibited growth, likely through disruption of cytoplasmic integrity, cytoplasmic membrane rupture, and inhibition of enzyme activity or synthesis (Bridges 1987, Cowan 1999, Hofstetter et al. 2005, Marei et al. 2012, Ullah et al. 2021). However, the magnitude of inhibition differed among monoterpene blends from the three lodgepole pine subspecies. The blend from subsp. *contorta* (British Columbia) was least inhibitory, followed by subsp. *latifolia* (Alberta), with the subsp. *murrayana* (Oregon) blend being most inhibitory. The stronger toxicity of the Alberta and Oregon blends is likely due to their higher overall concentration of monoterpenes, particularly the elevated concentrations of α-pinene, 3-carene, limonene, myrcene, and the phenylpropene 4-allylanisole (Cale et al. 2017, Chiu et al. 2017, Reid et al. 2017, Zhang et al. 2026). Terpinolene may also have contributed to the stronger inhibition observed under the Oregon and Alberta blends, as it occurred at higher concentrations in these blends and is particularly toxic to *G. clavigera* (Zhang et al. 2026). Oxygenated monoterpenes are particularly toxic because they disrupt intracellular redox homeostasis in fungal cells, leading to excessive accumulation of reactive oxygen species that inhibit energy production and suppress cell growth (Liang et al. 2023). Bornyl acetate, an oxygenated monoterpene, was most abundant in the Alberta blend, whereas 4-allylanisole was most abundant in the Oregon blend. The enrichment of these potentially antifungal compounds may have contributed to the inhibitory effects of the respective blends.

The two fungal species also differed in their growth responses, reflecting interspecific differences in monoterpene tolerance. *Grosmannia clavigera* exhibited greater tolerance than *O. montium*, consistent with its ability to dominate terpene-rich phloem (Agbulu et al. 2022, Zaman et al. 2025). *Grosmannia clavigera* employs multiple detoxification mechanisms (DiGuistini et al. 2011), including the expression of specialized ABC efflux transporter GcABC-G1 that limits intracellular monoterpene accumulation (Wang et al. 2013) and cytochrome P450-mediated oxygenation pathways (monooxygenases) that transform or deactivate toxic compounds (Lah et al. 2013, Chiu et al. 2019). Moreover, this fungus can utilize certain monoterpenes as a carbon source, especially (+)-limonene (Wang et al. 2014). *Grosmannia clavigera*’s enhanced growth under the British Columbia blend may reflect the utilization of (+)-limonene at low concentrations, whereas higher concentrations, as in the Alberta and Oregon blends, become inhibitory (Wang et al. 2014). These adaptations provide *G. clavigera* with a competitive advantage when colonizing new host trees, while it is unknown whether *O. montium* exhibits such adaptations. Consequently, *G. clavigera* is typically more virulent and forms longer lesions in host pines compared to *O. montium* (Rice et al. 2007b, Rice and Langor 2008).

Monoterpene composition and co-occurrence both influenced the emission of FVOCs. Fungal volatile production was most suppressed under the Oregon blend, except for relatively higher emissions of short-chain alcohols such as 3-methyl-1-butanol and 2-methyl-1-butanol. These fusel alcohols are produced from leucine and isoleucine catabolism through the Ehrlich pathway (Hazelwood et al. 2008). Their relative enrichment may reflect altered branched-chain amino acid utilization, nitrogen metabolism, or cellular redox balance under the Oregon blend. Fusel alcohols can also function as signalling molecules that influence fungal morphology, although their roles in *G. clavigera* and *O. montium* remain unknown.

Conversely, fungi under the Alberta blend, and *O. montium* under the Oregon blend, produced elevated concentrations of verbenone, an antiaggregation pheromone produced by MPB to prevent overcrowding on host trees (Pureswaran et al. 2000). When exposed to host defences, ophiostomatoid fungi can synthesize verbenone through α-pinene metabolism, potentially influencing beetle density regulation (Cale et al. 2019). This metabolic shift likely represents an evolved fungal strategy to reduce competition for host nutrients, indirectly benefiting both the fungi and MPBs. In addition*, O. montium* under the Alberta blend showed increased emissions of phenethyl acetate and *cis*-grandisol, both associated with MPB aggregation. While *cis*-grandisol production has been reported in *G. clavigera* (Wang et al. 2020b, Cale et al. 2016), its production by *O. montium* is novel. These findings suggest that host-derived monoterpenes may redirect fungal metabolism towards alternative secondary metabolic pathways, though the underlying mechanisms remain unclear. Together, the coemission of verbenone and aggregation-promoting compounds suggests a push–pull dynamic that modulates MPB colonization behaviour in relation to host chemical conditions (Kandasamy et al. 2016). In contrast to the Oregon blend, the Alberta blend appeared to impose only moderate stress, allowing sustained fungal growth while continuing to support fungal-beetle interactions.

Fungal volatile emissions were also differentially associated with fungal growth. The short-chain alcohols 2-methyl-1-butanol and isobutanol were positively correlated with fungal biomass. As products of branched-chain amino acid catabolism through the Ehrlich pathway, their abundance may reflect greater amino acid turnover in cultures with higher biomass rather than a direct effect of these compounds on fungal growth (Hazelwood et al. 2008). Their higher abundance in the British Columbia and control treatments, which also supported greater fungal growth, is consistent with this interpretation. In contrast, verbenone and *cis*-grandisol were negatively correlated with biomass, suggesting that their production was associated with culture conditions less favourable for fungal growth, although the underlying mechanisms remain unclear.

The observed species- and treatment-specific responses have important implications for MPB ecology. The symbiotic relationship between MPB and ophiostomatoid fungi depends on the fungus’s ability to metabolize host monoterpenes and influence beetle communication. *Grosmannia clavigera*’s higher tolerance and detoxification efficiency enhance MPB fitness by facilitating successful colonization of chemically defended trees (Zaman et al. 2023b, DiGuistini et al. 2011, Lah et al. 2013, Wang et al. 2014). Beetles carrying *G. clavigera* are typically larger than and more successful than those carrying *O. montium* (Bleiker and Six 2007, Agbulu et al. 2022). At the same time, *O. montium* appears to contribute more to signalling, particularly through verbenone production under moderate and high monoterpene stress (e.g. Alberta and Oregon blends), suggesting a regulatory role rather than a contribution to host colonization.

Collectively, our findings demonstrate that fungal growth and FVOC emissions vary across geographic locations within a single host species. These differences likely shape ecological interactions among beetles, fungi, and their hosts across diverse chemical environments. These findings further emphasize that *G. clavigera* and *O. montium* fulfill distinct but complementary functions within the MPB symbiosis: one facilitating colonization and detoxification, and the other modulating beetle behaviour. However, our experiments were conducted under simplified *in vitro* conditions using single isolates. Because PDB is more nutrient-rich than natural phloem, the magnitude of fungal growth and FVOC responses may differ under more nutritionally restricted conditions. *Grosmannia clavigera* consistently produced greater biomass than *O. montium* in single-species cultures and has shown greater growth and resource capture under similar conditions, suggesting that it may have contributed more to the mixed-culture biomass (Moore and Six 2015, Cale et al. 2016). In natural settings, monoterpene concentrations vary within and among trees, interact with other metabolites such as phenolics and tannins, and are influenced by dynamic fungal communities containing multiple strains and species. These interactions likely produce outcomes more complex than those observed here. Future research should therefore use species-specific molecular methods to clarify the contributions of each fungus in mixed cultures, examine these interactions under more natural conditions, identify the monoterpenes that most strongly regulate fungal metabolism, and elucidate the enzymatic mechanisms underlying their degradation or transformation. Understanding these processes under natural conditions will improve our ability to predict how symbiotic fungal communities respond to host chemical variation and, ultimately, how they influence MPB success and range expansion across diverse forest landscapes.

## Supplementary Material

fnag096_Supplemental_File
