# Supplementary material for: Pine monoterpenes drive growth, competition, and volatile metabolism of symbiotic fungi associated with the mountain pine beetle
Source: FEMS Microbiol Lett. 2026 Aug 26;373:fnag096. doi: 10.1093/femsle/fnag096 (PMC13548853; doi:10.1093/femsle/fnag096)
Supplement: Supplementary file 1 [file fnag096_supplemental_file.docx]

**Supplementary information**

**Pine Monoterpenes Drive Growth, Competition, and Volatile Metabolism of Symbiotic Fungi Associated with the Mountain Pine Beetle**

Table S1. Mean monoterpene concentrations (ng mg^-1^) fresh weight phloem tissue of lodgepole pine in three locations: Alberta, Canada; British Columbia, Canada; and Oregon, USA. Standard errors are reported after the ± sign.

| **Location** | British Columbia | Alberta | Oregon |
| --- | --- | --- | --- |
| **Subspecies** | *Pinus contorta* | | |
|  | subsp. *contorta* | subsp. *latifolia* | subsp. *murrayana* |
| (-)-$\alpha$-Pinene | 34.79 ± 5.72 | 85.21 ± 14.17 | 81.08 ± 10.19 |
| (+)-$\alpha$-Pinene | 14.79 ± 2.30 | 457.04 ± 117.69 | 38.82 ± 4.73 |
| (-)-$\beta$-Pinene | 6.16 ± 1.35 | 42.83 ± 7.89 | 37.77 ± 6.84 |
| (+)-$\beta$-Pinene | 5.28 ± 1.26 | 5.27 ± 1.18 | 2.02 ± 0.31 |
| (-)-Limonene | 37.76 ± 7.41 | 221.75 ± 55.70 | 227.60 ± 41.73 |
| (+)-Limonene | 15.28 ± 2.17 | 30.04 ± 3.49 | 32.95 ± 3.70 |
| (-)-Camphene | 52.48 ± 13.83 | 76.90 ± 10.79 | 56.73 ± 8.12 |
| (+)-Camphene | 18.42 ± 2.67 | 32.28 ± 4.77 | 34.02 ± 4.36 |
| 3-Carene | 157.65 ± 26.06 | 1006.78 ± 166.75 | 328.14 ± 44.57 |
| Linalool | 1.29 ± 0.17 | 0.84 ± 0.20 | 0.86 ± 0.17 |
| α-Terpinene | 4.98 ± 1.00 | 5.24 ± 1.26 | 2.57 ± 0.47 |
| γ-Terpinene | 4.53 ± 1.03 | 6.74 ± 0.76 | 3.88 ± 0.60 |
| Terpinolene | 34.58 ± 9.49 | 73.62 ± 12.77 | 112.24 ± 28.56 |
| 4-allylanisole | 7.43 ± 1.46 | 7.91 ± 1.24 | 38.77 ± 9.15 |
| (+)-Pulegone | 1.31 ± 0.19 | 0.94 ± 0.11 | 1.14 ± 0.17 |
| p-Cymene | 3.05 ± 0.47 | 2.69 ± 0.36 | 3.17 ± 0.50 |
| Bornyl acetate | 2.90 ± 0.56 | 27.57 ± 6.53 | 3.07 ± 1.03 |
| (-)-Borneol | 3.34 ± 0.56 | 3.48 ± 0.46 | 3.76 ± 0.63 |
| α-Phellandrene | 0.87 ± 0.11 | 0.60 ± 0.07 | 0.74 ± 0.10 |
| Ocimene | 12.86 ± 2.29 | 0.60 ± 0.06 | 11.72 ± 2.28 |
| Myrcene | 53.31 ± 7.16 | 100.20 ± 13.09 | 112.98 ± 13.52 |
| Sabinene hydrate | 3.78 ± 0.64 | 2.58 ± 0.31 | 7.99 ± 1.03 |
| Camphor | 2.44 ± 0.40 | 0.60 ± 0.12 | 0.56 ± 0.19 |
| α-Terpineol | 5.97 ± 0.88 | 1.87 ± 0.64 | 7.80 ± 2.71 |
| Thujone | 1.76 ± 0.18 | 0.76 ± 0.20 | 1.17 ± 0.28 |
| Total | 487.01 | 2194.34 | 1151.55 |

Table S2. The amount of each monoterpene (μL) was amended to 1mL of potato dextrose broth medium at three lodgepole pine locations: Alberta, Canada; British Columbia, Canada; and Oregon, USA. Ethanol was used as the solvent for dissolving monoterpenes. A total of 300 𝜇L monoterpene amendment was added to 30mL of liquid media for each sample.

| **Location** | British Columbia | Alberta | Oregon |
| --- | --- | --- | --- |
| **Subspecies** | *Pinus contorta* | | |
|  | subsp. *contorta* | subsp. *latifolia* | subsp. *murrayana* |
| (-)-$\alpha$-Pinene | 0.089 | 0.218 | 0.193 |
| (+)-$\alpha$-Pinene | 0.038 | 1.168 | 0.093 |
| (-)-$\beta$-Pinene | 0.016 | 0.106 | 0.089 |
| (+)-$\beta$-Pinene | 0.013 | 0.013 | 0.005 |
| (-)-Limonene | 0.099 | 0.593 | 0.553 |
| (+)-Limonene | 0.040 | 0.078 | 0.080 |
| (-)-Camphene | 0.139 | 0.200 | 0.138 |
| (+)-Camphene | 0.048 | 0.084 | 0.083 |
| 3-Carene | 0.400 | 2.551 | 0.777 |
| Linalool | 0.003 | 0.002 | 0.002 |
| α-Terpinene | 0.013 | 0.014 | 0.006 |
| γ-Terpinene | 0.012 | 0.017 | 0.009 |
| Terpinolene | 0.089 | 0.187 | 0.266 |
| 4-allylanisole | 0.017 | 0.018 | 0.082 |
| (+)-Pulegone | 0.003 | 0.002 | 0.002 |
| p-Cymene | 0.008 | 0.007 | 0.008 |
| Bornyl acetate | 0.006 | 0.060 | 0.006 |
| (-)-Borneol | 0.007 | 0.007 | 0.008 |
| α-Phellandrene | 0.002 | 0.001 | 0.002 |
| Ocimene | 0.035 | 0.002 | 0.030 |
| Myrcene | 0.148 | 0.278 | 0.292 |
| Sabinene hydrate | 0.008 | 0.005 | 0.016 |
| Camphor | 0.005 | 0.001 | 0.001 |
| α-Terpineol | 0.014 | 0.004 | 0.017 |
| Thujone | 0.004 | 0.002 | 0.003 |
| Total monoterpenes | 1.258 | 5.620 | 2.760 |
| Volume ethanol | 8.742 | 4.380 | 7.240 |
| Volume total | 10 | 10 | 10 |

Table S3. Post-hoc pairwise comparisons of fungal biomass (mg) among treatments, using Dunn’s test following a Kruskal–Wallis test. Each treatment represents the interactive effects of a fungal culture × a monoterpene amendment. Fungal treatments are *Grosmannia clavigera* (Gc), *Ophiostoma montium* (Om), and mixed-species culture (Gc & Om). Z-scores indicate the standardized differences between groups. Statistically significant differences are indicated by adjusted p-value < 0.05.

| **Comparison** | **Z-score** | **Unadjusted P-value** | **Adjusted P-value** |
| --- | --- | --- | --- |
| Alberta Gc/Alberta Gc & Om | 1.72 | 0.09 | 0.14 |
| Alberta Gc/Alberta Om | 4.26 | 2.03E-05 | 0.00 |
| Alberta Gc & Om/Alberta Om | 2.55 | 0.01 | 0.03 |
| Alberta Gc/British Columbia Gc | -1.62 | 0.11 | 0.17 |
| Alberta Gc & Om/British Columbia Gc | -3.34 | 0.00 | 0.00 |
| Alberta Om/British Columbia Gc | -5.88 | 4.06E-09 | 1.07E-07 |
| Alberta Gc/British Columbia Gc & Om | 0.26 | 0.79 | 0.83 |
| Alberta Gc & Om/British Columbia Gc & Om | -1.46 | 0.15 | 0.22 |
| Alberta Om/British Columbia Gc & Om | -4.00 | 6.31E-05 | 0.00 |
| British Columbia Gc/British Columbia Gc & Om | 1.88 | 0.06 | 0.11 |
| Alberta Gc/British Columbia Om | 3.73 | 0.00 | 0.00 |
| Alberta Gc & Om/British Columbia Om | 2.01 | 0.04 | 0.08 |
| Alberta Om/British Columbia Om | -0.54 | 0.59 | 0.67 |
| British Columbia Gc/British Columbia Om | 5.35 | 9.00E-08 | 1.58E-06 |
| British Columbia Gc & Om/British Columbia Om | 3.47 | 0.00 | 0.00 |
| Alberta Gc/Control Gc | -1.80 | 0.07 | 0.12 |
| Alberta Gc & Om/Control Gc | -3.51 | 0.00 | 0.00 |
| Alberta Om/Control Gc | -6.06 | 1.39E-09 | 4.86E-08 |
| British Columbia Gc/Control Gc | -0.18 | 0.86 | 0.89 |
| British Columbia Gc & Om/Control Gc | -2.06 | 0.04 | 0.08 |
| British Columbia Om/Control Gc | -5.52 | 3.37E-08 | 7.08E-07 |
| Alberta Gc/Control Gc & Om | -0.22 | 0.83 | 0.86 |
| Alberta Gc & Om/Control Gc & Om | -1.93 | 0.05 | 0.10 |
| Alberta Om/Control Gc & Om | -4.48 | 7.55E-06 | 5.29E-05 |
| British Columbia Gc/Control Gc & Om | 1.40 | 0.16 | 0.24 |
| British Columbia Gc & Om/Control Gc & Om | -0.48 | 0.63 | 0.70 |
| British Columbia Om/Control Gc & Om | -3.94 | 8.09E-05 | 0.00 |
| Control Gc/Control Gc & Om | 1.58 | 0.11 | 0.18 |
| Alberta Gc/Control Om | 0.65 | 0.51 | 0.59 |
| Alberta Gc & Om/Control Om | -1.06 | 0.29 | 0.36 |
| Alberta Om/Control Om | -3.61 | 0.00 | 0.00 |
| British Columbia Gc/Control Om | 2.27 | 0.02 | 0.05 |
| British Columbia Gc & Om/Control Om | 0.39 | 0.70 | 0.75 |
| British Columbia Om/Control Om | -3.07 | 0.00 | 0.01 |
| Control Gc/Control Om | 2.45 | 0.01 | 0.03 |
| Control Gc & Om/Control Om | 0.87 | 0.39 | 0.47 |
| Alberta Gc/Ethanol Gc | -0.53 | 0.60 | 0.67 |
| Alberta Gc & Om/Ethanol Gc | -2.24 | 0.02 | 0.05 |
| Alberta Om/Ethanol Gc | -4.79 | 1.67E-06 | 1.76E-05 |
| British Columbia Gc/Ethanol Gc | 1.09 | 0.27 | 0.36 |
| British Columbia Gc & Om/Ethanol Gc | -0.79 | 0.43 | 0.51 |
| British Columbia Om/Ethanol Gc | -4.25 | 2.11E-05 | 0.00 |
| Control Gc/Ethanol Gc | 1.27 | 0.20 | 0.29 |
| Control Gc & Om/Ethanol Gc | -0.31 | 0.76 | 0.80 |
| Control Om/Ethanol Gc | -1.18 | 0.24 | 0.33 |
| Alberta Gc/Ethanol Gc & Om | 0.64 | 0.52 | 0.60 |
| Alberta Gc & Om/Ethanol Gc & Om | -1.08 | 0.28 | 0.36 |
| Alberta Om/Ethanol Gc & Om | -3.62 | 0.00 | 0.00 |
| British Columbia Gc/Ethanol Gc & Om | 2.26 | 0.02 | 0.05 |
| British Columbia Gc & Om/Ethanol Gc & Om | 0.38 | 0.70 | 0.76 |
| British Columbia Om/Ethanol Gc & Om | -3.09 | 0.00 | 0.01 |
| Control Gc/Ethanol Gc & Om | 2.43 | 0.01 | 0.03 |
| Control Gc & Om/Ethanol Gc & Om | 0.86 | 0.39 | 0.47 |
| Control Om/Ethanol Gc & Om | -0.01 | 0.99 | 0.99 |
| Ethanol Gc/Ethanol Gc & Om | 1.17 | 0.24 | 0.33 |
| Alberta Gc/Ethanol Om | 1.80 | 0.07 | 0.12 |
| Alberta Gc & Om/Ethanol Om | 0.09 | 0.93 | 0.94 |
| Alberta Om/Ethanol Om | -2.46 | 0.01 | 0.03 |
| British Columbia Gc/Ethanol Om | 3.42 | 0.00 | 0.00 |
| British Columbia Gc & Om/Ethanol Om | 1.54 | 0.12 | 0.19 |
| British Columbia Om/Ethanol Om | -1.92 | 0.05 | 0.10 |
| Control Gc/Ethanol Om | 3.60 | 0.00 | 0.00 |
| Control Gc & Om/Ethanol Om | 2.02 | 0.04 | 0.08 |
| Control Om/Ethanol Om | 1.15 | 0.25 | 0.33 |
| Ethanol Gc/Ethanol Om | 2.33 | 0.02 | 0.04 |
| Ethanol Gc & Om/Ethanol Om | 1.16 | 0.24 | 0.32 |
| Alberta Gc/Oregon Gc | 3.01 | 0.00 | 0.01 |
| Alberta Gc & Om/Oregon Gc | 1.30 | 0.19 | 0.28 |
| Alberta Om/Oregon Gc | -1.25 | 0.21 | 0.30 |
| British Columbia Gc/Oregon Gc | 4.63 | 3.57E-06 | 2.89E-05 |
| British Columbia Gc & Om/Oregon Gc | 2.75 | 0.01 | 0.02 |
| British Columbia Om/Oregon Gc | -0.71 | 0.48 | 0.56 |
| Control Gc/Oregon Gc | 4.81 | 1.51E-06 | 1.76E-05 |
| Control Gc & Om/Oregon Gc | 3.23 | 0.00 | 0.00 |
| Control Om/Oregon Gc | 2.36 | 0.02 | 0.04 |
| Ethanol Gc/Oregon Gc | 3.54 | 0.00 | 0.00 |
| Ethanol Gc & Om/Oregon Gc | 2.38 | 0.02 | 0.04 |
| Ethanol Om/Oregon Gc | 1.21 | 0.23 | 0.32 |
| Alberta Gc/Oregon Gc & Om | 2.89 | 0.00 | 0.01 |
| Alberta Gc & Om/Oregon Gc & Om | 1.18 | 0.24 | 0.33 |
| Alberta Om/Oregon Gc & Om | -1.37 | 0.17 | 0.25 |
| British Columbia Gc/Oregon Gc & Om | 4.51 | 6.37E-06 | 4.78E-05 |
| British Columbia Gc & Om/Oregon Gc & Om | 2.63 | 0.01 | 0.02 |
| British Columbia Om/Oregon Gc & Om | -0.83 | 0.41 | 0.48 |
| Control Gc/Oregon Gc & Om | 4.69 | 2.75E-06 | 2.62E-05 |
| Control Gc & Om/Oregon Gc & Om | 3.11 | 0.00 | 0.01 |
| Control Om/Oregon Gc & Om | 2.24 | 0.03 | 0.05 |
| Ethanol Gc/Oregon Gc & Om | 3.42 | 0.00 | 0.00 |
| Ethanol Gc & Om/Oregon Gc & Om | 2.25 | 0.02 | 0.05 |
| Ethanol Om/Oregon Gc & Om | 1.09 | 0.28 | 0.35 |
| Oregon Gc/Oregon Gc & Om | -0.12 | 0.90 | 0.92 |
| Alberta Gc/Oregon Om | 4.67 | 2.96E-06 | 2.59E-05 |
| Alberta Gc & Om/Oregon Om | 2.96 | 0.00 | 0.01 |
| Alberta Om/Oregon Om | 0.41 | 0.68 | 0.74 |
| British Columbia Gc/Oregon Om | 6.29 | 3.10E-10 | 1.63E-08 |
| British Columbia Gc & Om/Oregon Om | 4.41 | 1.02E-05 | 6.69E-05 |
| British Columbia Om/Oregon Om | 0.95 | 0.34 | 0.42 |
| Control Gc/Oregon Om | 6.47 | 9.87E-11 | 1.04E-08 |
| Control Gc & Om/Oregon Om | 4.89 | 1.01E-06 | 1.33E-05 |
| Control Om/Oregon Om | 4.02 | 5.78E-05 | 0.00 |
| Ethanol Gc/Oregon Om | 5.20 | 1.98E-07 | 2.97E-06 |
| Ethanol Gc & Om/Oregon Om | 4.03 | 5.47E-05 | 0.00 |
| Ethanol Om/Oregon Om | 2.87 | 0.00 | 0.01 |
| Oregon Gc/Oregon Om | 1.66 | 0.10 | 0.16 |
| Oregon Gc & Om/Oregon Om | 1.78 | 0.08 | 0.13 |
